# Compromised diabetic heart function is not affected by miR-378a upregulation upon hyperglycemia

**DOI:** 10.1007/s43440-023-00535-8

**Published:** 2023-10-18

**Authors:** Urszula Florczyk-Soluch, Katarzyna Polak, Reece Sabo, Alicja Martyniak, Jacek Stępniewski, Józef Dulak

**Affiliations:** Department of Medical Biotechnology, Faculty of Biochemistry, Biophysics and Biotechnology, Jagiellonian University, Gronostajowa 7, 30-387 Kraków, Poland

**Keywords:** MicroRNA-378a, Diabetic cardiomyopathy, Streptozotocin, Human induced pluripotent stem cells

## Abstract

**Background:**

Cardiac-abundant microRNA-378a (miR-378a) is associated with postnatal repression of insulin–like growth factor 1 receptor (IGF-1R) controlling physiological hypertrophy and survival pathways. IGF-1/IGF-1R axis has been proposed as a therapeutic candidate against the pathophysiological progress of diabetic cardiomyopathy (DCM). We ask whether hyperglycemia-driven changes in miR-378a expression could mediate DCM progression.

**Methods:**

Diabetes mellitus was induced by streptozotocin (STZ) (55 mg/kg i.p. for 5 days) in male C57BL/6 wild type (miR-378a+/+) and miR-378a knockout (miR-378a−/−) mice. As a parallel human model, we harnessed human induced pluripotent stem cell-derived cardiomyocytes (hiPSC-CM miR378a+/+ vs. hiPSC-CM miR378a−/−) subjected to high glucose (HG) treatment.

**Results:**

We reported miR-378a upregulation in cardiac diabetic milieu arising upon STZ administration to wild-type mice and in HG-treated hiPSC-CMs. Pro-hypertrophic IGF-1R/ERK1/2 pathway and hypertrophic marker expression were activated in miR-378a deficiency and upon STZ/HG treatment of miR-378a+/+ specimens in vivo and in vitro suggesting miR-378a-independent hyperglycemia-promoted hypertrophy. A synergistic upregulation of IGF-1R signaling in diabetic conditions was detected in miR-378a−/− hiPSC-CMs, but not in miR-378a−/− hearts that showed attenuation of this pathway, pointing to the involvement of compensatory mechanisms in the absence of miR-378a. Although STZ administration did not cause pro-inflammatory or pro-fibrotic effects that were detected in miR-378a−/− mice, the compromised diabetic heart function observed in vivo by high-resolution ultrasound imaging upon STZ treatment was not affected by miR-378a presence.

**Conclusions:**

Overall, data underline the role of miR-378a in maintaining basal cardiac structural integrity while pointing to miR-378a-independent hyperglycemia-driven cardiac hypertrophy and associated dysfunction.

**Supplementary Information:**

The online version contains supplementary material available at 10.1007/s43440-023-00535-8.

## Introduction

Rising from the long subclinical period, diabetic cardiomyopathy (DCM) proceeds in the systolic dysfunction stage, becoming a chronic cardiac complication eventually leading to heart failure [1]. Sustained cardiomyocyte hypertrophy and excessive interstitial fibrosis lay at the pathophysiological roots of DCM [2, 3]. The causal molecular background is associated with metabolic disturbances (hyperglycemia) [1, 4], however, in long-term randomized trials normalization of blood glucose levels in diabetic patients did not halt DCM progression [5].

Whether diabetes-related alterations of cardiac miRNAs (miRs) landscape may contribute to the pathogenesis of DCM is of growing interest [1, 5]. Being one of the cardiac-enriched miRs, miRNA-378a exhibits cardiomyocyte-selective expression while is not detected in non-muscle cells [6–8]. It is located in the first intron of the *Ppargc1b* gene, encoding PGC-1β, a key regulator of energy metabolism, and gives rise to two functional mature strands, miR-378a-3p and miR-378a-5p [9]. Pro-angiogenic and anti-inflammatory activity of miR-378a was reported by us recently in murine skeletal muscles [6].

Anti-hypertrophic action of miR-378a was demonstrated in cardiac in vitro model of phenylephrine-induced cardiomyocyte hypertrophy and in an in vivo model of cardiac hypertrophy (pressure overload by thoracic aortic constriction) [8, 10] but has not examined so far in the context of diabetes. In vitro, in rat primary cardiomyocytes miR-378a inhibited phenylephrine-induced hypertrophy and repressed pro-hypertrophic mitogen-activated protein kinase (MAPK) signaling at four levels, targeting insulin-like growth factor-1 (IGF-1) receptor (IGF-1R) [7] and downstream mediators: MAPK1, GRB2 and KSR1 [10]. Our recent study indicates also miR-378a-dependent regulation of hypertrophic growth of human induced pluripotent stem cell-derived cardiomyocytes (hiPSC-CMs) [11]. In addition, previous data point to anti-fibrotic effects of miR-378a in the murine model of pressure overload [8, 10].

MiR-378a expression is detected at a relatively low level in the fetal heart, progressively increasing after birth and continuing in aged mice, which is associated with postnatal repression of cardiac IGF-1R [7]. Closing a miR-378a/ IGF-1R/IGF-1 feedback loop, IGF-1 may act as an inhibitor of miR-378a triggering cell survival signals in the heart [7]. The level of IGF-1 is decreased in the serum of animals and patients with diabetes [12]. Accordingly, in the diabetic environment a strategy of IGF-1/IGF-1R overexpression ameliorated the pathophysiological progress of DCM through antioxidative and anti-inflammatory processes [12–14]. Here, we consider the involvement of miR-378a in the pathogenesis of DCM with particular emphasis on the regulation of IGF-1R signaling.

## Materials and methods

### hiPSC-CMs

hiPSCs were generated from healthy male donor peripheral blood mononuclear cells (agreement from Jagiellonian University Bioethical Committee no. 122.6120.303.2016) using CytoTune™-iPS 2.0 Sendai Reprogramming Kit (ThermoFisher Scientific, cat.no. A16517) as reported by us previously [11]. Cells were cultured in standard conditions (37 °C, 5% CO_2_) on Geltrex-coated plates in Essential 8 Basal Medium with supplement (Gibco, cat.no. A15169-01/A15171-01) and passaged with 0.5 mM EDTA at ~ 80% confluency.

Cardiac differentiation of hiPSCs was performed using small molecules regulating the WNT pathway, as described previously [11]. Upon differentiation cells were detached with TrypLE Express Enzyme (ThermoFisher Scientific, cat. no. 12604021), harvested in RPMI-1640 Medium (Sigma–Aldrich, cat.no. R8758) containing 20% FBS, and centrifuged (200 × *g*, 5 min). hiPSC-CMs were resuspended in RPMI-1640 medium containing 2% B-27 supplement (ThermoFisher Scientific, cat. no. 17504001) (RPMI 1640/B-27) and seeded on Geltrex-coated plates.

### High glucose treatment

hiPSC-CMs were cultured for 48 h in increased concentration of glucose (33 mM, 55 mM, Sigma, cat.no. G7021) in RPMI 1640/B-27 medium (with basal 11 mM glucose). Mannitol (55 mM) was used as an osmotic control.

### Mice

miR-378–/– mice at 129SvEv/C57BL/6 background were the gift of Eric Olson (Department of Molecular Biology, University of Texas Southwestern Medical Center, Dallas, Texas, USA) [15]. Mice were backcrossed with C57Bl/6J mice for ten generations to obtain animals on a clear C57BL/6 background. Animal experiments were performed on male mice after approval (No. 108/2021) by the second Institutional Animal Care and Use Committee (IACUC) in Krakow, Poland, according to Polish and European legislation. Mice were housed under specific pathogen-free (SPF) conditions in individually ventilated cages with a 14 h/10 h light/dark cycle and were kept on a normal, chow diet with water and food available ad libitum. For fasting mice were transferred for 4 h to new cages without food, with water ad libitum.

### STZ injections and material collection

Type 1 diabetes was induced in 10–13-week-old miR-378a−/− mice (C57Bl6 background) and age-matched wild-type counterparts (miR-378a+/+) by STZ treatment. STZ (55 mg/kg b.w., Sigma–Aldrich, cat.no. S0130) dissolved in 0.1 mol/l citrate buffer (pH 4.5) or citrate buffer alone was administered to mice (5.5 µL/ 1 g) via intraperitoneal injections (i.p.) for five consecutive days [14]. Blood glucose level was measured upon 4 h-starvation using AccuCheck glucometer (Roche Diagnostics) 4 and 8 weeks after the last STZ injection. Mice were considered hyperglycemic when their blood glucose level was a minimum 240 mg/dL. Upon 8 weeks of STZ treatment, the assessment of cardiac parameters in vivo by high-resolution ultrasound imaging was performed and the mice were sacrificed thereafter. Peripheral blood was immediately collected, and hearts were perfused with heparinized saline (0.5 IU/ml) and potassium chloride (30 mM) to arrest heart contraction in diastole.

### Visualization and quantification of heart function by ultrasonography

For the assessment of cardiac parameters in vivo high-resolution ultrasound imaging system for the examination of small animals (Vevo 2100, Visual Sonics) with an MS-400: 38 MHz MicroScan transducer was used. Hair from the chest was removed the day before scanning. Anesthesia was induced in 3% isoflurane (Aerrane, Baxter)/O2 filled induction chamber. Once anesthetized, the mouse was placed dorsally on heated platform and anesthesia was redirected to the nose cone (maintained at 1.5–2.0% isoflurane, 2.5 L/min). Each paw pad was taped down to platform on ultrasonic gel dots for ECG readings. Pre‐warmed ultrasonic gel was applied to the shaved spot. The heart was observed using parasternal long axis (PSLAX) view at first and upon 90 clockwise rotation of the probe short axis (SAX) view was captured with M mode line on the center of the LV. Heart rate was kept between 300‐600 BPM. For quantification of cardiac function anterior and posterior wall edge were traced through 3–4 beats on 2–4 independent tracings.

### RNA-based analysis

Dissected cardiac tissues were collected into RNA*later* tissue storage reagent (Sigma–Aldrich, cat.no. R0901), frozen in liquid nitrogen, and stored at – 80 °C. Samples were homogenized in phenol/guanidine-based QIAzol Lysis Reagent (Qiagen, cat.no.79306). Total RNA was isolated by organic extraction upon the addition of chloroform.

Total RNA isolation from hiPSC-CMs was done using Fenozol reagent (A&A Biotechnology, cat. no. 203–100) with chloroform. Upon isopropanol-based precipitation of RNA from the aqueous phase and ethanol washing, the RNA pellet was resuspended in RNase-free water. RNA concentration and quality were determined by NanoDrop Spectrophotometer (ThermoFisher Scientific).

Total RNA was used for reverse transcription (RT) of either mRNA (500 ng) or miRNA (10 ng) performed with RevertAid reverse transcriptase (200 U/μL, Thermo Fisher Scientific, cat. no. EP0442) and miRCURY LNA RT kit (Qiagen, cat. no. 339340), respectively, carried out in ProFlex PCR System (ThermoFisher Scientific). Gene expression analysis and miRNA detection were done using quantitative PCR (qPCR) as described previously [6] based on SYBR Green JumpStart Taq Ready Mix (Sigma–Aldrich, cat.no. S4438) (Table 1) or miRCURY LNA SYBR Green PCR kit (Qiagen, cat. no. 339346) (Table 2), respectively, carried out in StepOne Plus Real-Time PCR System (Applied Biosystems) and analyzed with StepOne Software v2.3. Relative quantification was based on the 2^–Δ*Ct*^ method with *Eef2, Actb,* or U6 snRNA (for miRNA) as a reference.

**Table 1:** The sequences of primers used for the determination of gene expression at mRNA level by qPCR

*Actb*	F—5′-TCCTTCGTTGCCGGTCCACA-3′
	R—5′-GCTTTGCACATGCCGGAGCC-3′
*Igf1r*	F—5′-TTCTTCTCTTCATCGCCGCAGACT-3′
	R—5′-ATCGCGATTTCTGCGCCAACA-3′
*Mapk1*	F—5′-CGTGTTGCAGATCCAGACCATGAT-3′
	R—5′-TGGACTTGGTGTAGCCCTTGGAA-3′
*Pik3ca*	F—5′-CCAACGGACAGTGCTCCTCCTT-3′
	R—5′-TCCTGATCTTCCTCGTGCTGCG-3′
*Nppa*	F—5′-CCGGTACCGAAGATAACAGC-3′
	R—5′-CTCCAGGAGGGTATTCACCA-3′
*Nppb*	F—5′-GCCATTTCCTCCGACTTTTCTC-3′
	R—5′-GAGGTCACTCCTATCCTCTGG-3′
*Myh7*	F—5′-CCGAGTCCCAGGTCAACAA-3′
	R—5′-CTTCACGGGCACCCTTGGA-3′
*Col1a1*	F—5′-CGATCCAGTACTCTCCGCTCTTCC-3′
	R—5′-ACTACCGGGCCGATGATGCTAACG-3′
*Fn1*	F—5′-AGCCTGCTCATCAGTTGGGA-3′
	R—5′-GATGGAAACTGGCTTGCTGC-3′
*Tnfa*	F—5′-GACCCTCACACTCAGATCATC-3′
	R—5′-CCACTTGGTGGTTTGCTACGA-3′
*Il1b*	F—5′-CCGACAGCACGAGGCTTT-3′
	R—5′-CTGGTGTGTGACGTTCCCATT-3′
*Il6*	F—5′-AAAGAGTTGTGCAATGGCAAT-3′
	R-5′-AAGTGCATCATCGTTGTTCAT-3′
*Vegf*	F—5′-ATGCGGATCAAACCTCACCAA-3′
	R—5′-TTAACTCAAGCTGCCTCGCCT-3′
*EEF2*	F—5′-TCAGCACACTGGCATAGAGGC-3′
	R—5′-GACATCACCAAGGGTGTGCAG-3′
*IGF1R*	R—5′-AATGAAGTCTGGCTCCGGAGGAGGGTC-3′
	R—5′-TGCTGATAGTCGTTGCGGATGTCGATG-3′
*MAPK1*	F—5′-CGTGTTGCAGATCCAGACCATGAT-3′
	R—5′-TGGACTTGGTGTAGCCCTTGGAA-3′

**Table 2:** The sequences of primers used for the determination of miRNA level using miRCURY LNA PCR kit

U6 snRNA	5′ CGCAAGGATGACACGCAAATTC 3′
miR-378a-3p	5′ ACUGGACUUGGAGUCAGAAGG 3′
miR-378a-5p	5′ CUCCUGACUCCAGGUCCUGUGU 3′

### Western blot

Whole protein lysates were obtained from dissected cardiac tissues (pre-frozen in liquid nitrogen and stored at − 80 °C) upon homogenization in 500 μL of ice-cold 1% Triton X100 in PBS and proteinase inhibitors (Roche Diagnostic) using TissueLyser (Qiagen). Proteins from hiPSC-CMs were isolated using Pierce RIPA Buffer (Thermo Scientific, cat. no. 89900) with proteinase inhibitors. Protein samples (30 μg) were subjected to SDS–PAGE and Western Blot under conditions specified previously [11] using primary antibodies against IGF-1R (1:500, Santa Cruz Biotechnology, cat. no. sc-713), PathScan^®^ Multiplex Western Cocktail I: phospho-AKT (Ser473), phospho-p44/42 MAPK (ERK1/2, Thr202/Tyr204) (1:1000, Cell Signaling, cat. no. 5301), AKT (1:500, Cell Signaling, cat. no. 9272S), p44/42 MAPK (ERK1/2) (1:500, Cell Signaling, cat. no. 9102), GAPDH (1:1000, Santa Cruz Biotechnology, cat. no. sc-59540) and HRP-conjugated secondary antibodies: anti-rabbit (1:5000, Cell Signaling, cat. no. 7074P2), anti-mouse (1:10,000, BD Pharmingen, cat. no. 554002). Antibodies were prepared in a blocking buffer containing 5% BSA (bovine serum albumin) in 0.1% Tween 20 in TBS.

#### ELISA

The protein level of FGF2, VEGFA, and TGF-ß in heart lysates was assessed by ELISA (R&D Systems. cat.no. DY3139, cat.no. D4493, cat.no. DY1679, respectively) according to vendor’s protocol.

### Immunofluorescence (IF) of hiPSC-CMs

IF stainings were performed as described previously [11]. Cells washed with PBS were fixed with 4% paraformaldehyde (15 min), permeabilized with 0.1% Triton X-100 in PBS (15 min), and blocked in 3% BSA in PBS (30 min) with PBS rinsing steps in between. Primary antibody against α-actinin (mouse anti-human, 1:300, overnight, 4 °C, Sigma–Aldrich, cat.no. A7811) and secondary donkey anti-mouse AlexaFluor488 antibody (1:400, 1 h, Invitrogen, cat. no. A21202) supplemented with Hoechst33342 (1 μg/mL, Sigma–Aldrich, cat. no. 14533) were diluted in blocking buffer. Upon PBS washing steps pictures were taken with Leica DMi8 microscope with CMOS Leica MC170 HD camera and Leica Application Suite X (Las X) Life Science microscope software platform. Cell area was calculated by the blinded investigator using ImageJ software.

### Histological procedures and IF of paraffin cardiac sections

Hearts were fixed for 48 h in 10% formalin and paraffin-embedded. Paraffin sections cut with the automatic microtome (ThermoFisher Scientific) (8 μm-thick) were deparaffinized and subjected to hematoxylin and eosin (H&E) staining to visualize tissue morphology, inflammatory infiltration, and necrosis, Masson’s Trichrome staining to assess fibrosis, according to the vendor’s instructions (Sigma–Aldrich), and IF stainings. For IF, a previously established protocol was used with modifications [16] described in detail in Supplementary material.

Pictures were taken with Carl-Zeiss LSR-510 meta laser scanning confocal microscope or Leica DMi8 microscope with CMOS Leica MC170 HD camera with Leica Application Suite X (Las X) Life Science microscope software platform.

Semi-quantitative analysis of leukocyte infiltration and necrotic events (H&E) was performed within each scan of the whole heart cross-section (1 scan per mouse) from all fields of view (~ 50 images per scan) and expressed as the average arbitrary unit per field of view. Quantitative analysis of collagen area (Masson’s Trichrome) was performed from each scan of the whole heart cross-section using QuPath-0.2.3 software and expressed as the % of collagen area per total heart area. α-SMA-positive vessels were counted from all fields of view (35–50 images per scan) within each scan of the whole heart section and expressed as the average number of arterioles per field of view.

### Blood cell count

The peripheral blood from the eye was collected directly into EDTA-coated tubes and was analyzed by the ABC Vet device (Horiba ABX).

### Colorimetric evaluation of serum CK and LDH

The eye blood collected into the EPP tube was allowed to clot at room temperature for 30 min. Serum was obtained upon centrifugation (1000 × *g*, 10 min, 4 °C). Serum creatine kinase (CK) and lactate dehydrogenase (LDH) activity was measured using the diagnostic Liquick Cor-CK kit (cat. no. 1–219) and Liquick Cor-LDH kit (cat. no. 1–239), respectively, according to the vendor’s protocol (Cormay).

### Statistics

Numerical data are presented as mean ± SEM. The outliers were excluded based on Grubb’s test. One-way ANOVA (for one variable comparison) or two-way ANOVA (multiple group comparisons), followed by Tukey’s post hoc test was used as primary tests to determine statistical significance (*p* < 0.05). Student’s *t* test was used additionally for a direct comparison of two groups that were not significantly different using ANOVA tests. GraphPad 8™ software was used for all statistical analysis. *p* values 0.05–0.1 were considered as a statistical tendency.

The values of statistics (main effects and interactions) were provided where applicable (for ANOVA: *F*, *df*, and *p* values; for Student’s *t* test: *t*, *df*, and *p* values). The *n* number indicating the number of animals per group or in vitro experimental repetitions as well as the specific statistical test used with associated markings are underlined in the figure captions.

## Results

### STZ administration induces hyperglycemia, lymphopenia, and monocytopenia

**Fig. 1:**
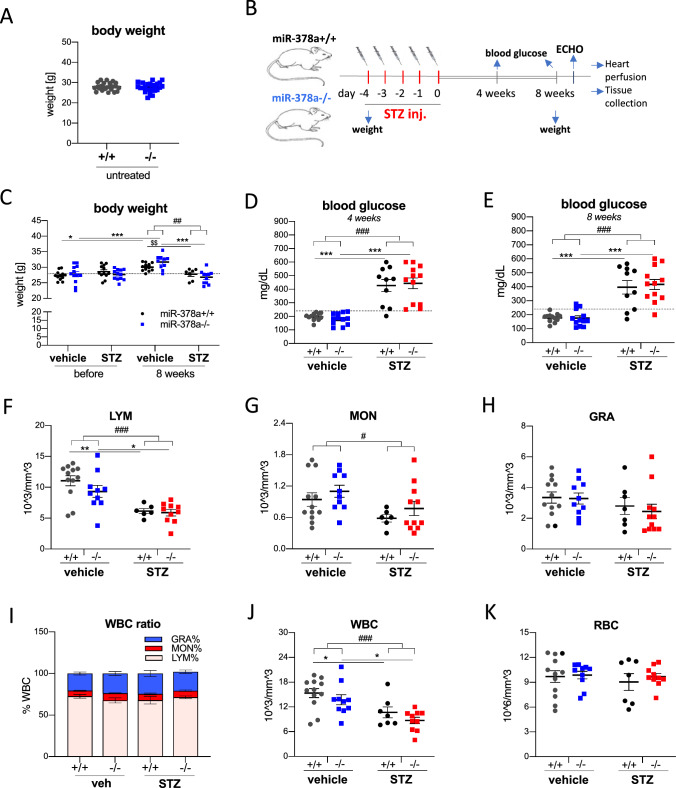
STZ treatment induces hyperglycemia, leukopenia, and lymphopenia in the blood. **A** Body weight of untreated miR-378a−/− and miR-378a+/+ mice (10–13-week-old) (*n* = 24), **B** Scheme of the experimental procedure, and **C** body weight (*n* = 7–12) 8 weeks upon streptozotocin (STZ) treatment. **D**, **E** Blood glucose level upon 4 h starvation **D** 4 weeks and **E** 8 weeks after the last STZ injection (*n* = 10–12). **F** lymphocytes (LYM), **G** monocytes (MON), **H** granulocytes (GRA), **I** white blood cells (WBC) ratios, **J** WBC and **K** red blood cells (RBC) in serum at week 8 upon STZ administration. Blood cell count (*n* = 7–12). Data are presented as mean ± SEM. ^#^*p* < 0.05, ^###^*p* < 0.001—two-way ANOVA variation; *p < 0.05, **p < 0.01, ***p < 0.001 by two-way ANOVA with Tukey’s post hoc test; ^$$^*p* < 0.01 by unpaired two-tailed Student’s *t* test

As reported by us recently [17], the body weight of untreated miR-378a−/− and miR-378+/+ mice was comparable (*t*_46_ = 0.3838, *p* > 0.1, Fig. 1A). STZ administration, as one of chemically induced type 1 diabetes models relates to the destruction of the pancreatic beta cells leading to deficiency in insulin production, hyperglycemia, and weight loss [18]. We observed a significant effect of STZ on body weight at 8 weeks upon 5-day treatment as shown by a two-way ANOVA (*F*_1,36_ = 35.12, *p* < 0.0001). A significant increase in weight was detected in vehicle-treated miR-378a+/+ (*p* = 0.024) and miR-378a−/− (*p* = 0.0005) mice, while in response to STZ treatment, we observed the lack of weight gain in time in both genotypes (Fig. 1B, C). Concomitantly blood glucose level increased already at week 4 (*F*_1,42_ = 70.80, *p* < 0.0001, Fig. 1D) and week 8 (*F*_1,42_ = 59.42, *p* < 0.0001, Fig. 1E) upon STZ administration with no difference between miR-378a−/− and miR-378+/+ mice.

Accordingly, STZ-treated animals of both genotypes showed a decreased number of lymphocytes (*F*_1,34_ = 26.03, *p* < 0.0001, Fig. 1F) and monocytes (*F*_1,35_ = 6.688, *p* < 0.0140, Fig. 1G), but not granulocytes (*F*_1,36_ = 2.535, *p* = 0.12, Fig. 1H) proving immunosuppressive action of STZ. The percentage of each of those populations (Fig. 1I) did not change significantly in response to STZ while the total WBC density decreased (*F*_1,35_ = 18.28, *p* < 0.0001, Fig. 1I). The number of red blood cells was not affected by STZ treatment (*F*_1,35_ = 0.4123, *p* = 0.5250, Fig. 1K).

### miR-378a is upregulated in hyperglycemic murine heart

**Fig. 2:**
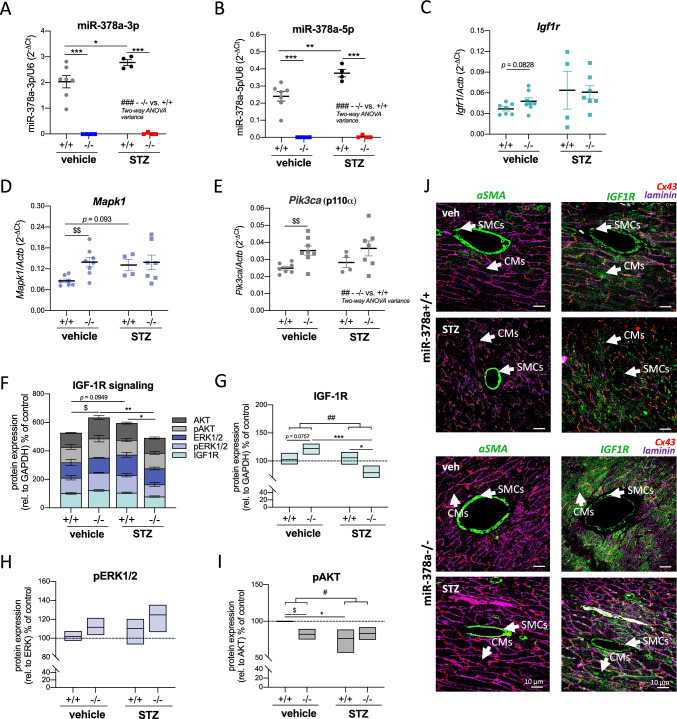
miR-378a is upregulated in hyperglycemic murine heart. **A**–**I** Heart lysates and **J** cardiac histological sections were obtained from either control or STZ-treated miR-378a+/+ and miR-378a−/− mice. The expression of **A** miR-378a-3p and **B** miR-378a-5p is increased in heart lysates of miR-378a+/+ mice 8 weeks upon STZ treatment, LNA miRCURY qPCR (*n* = 4–8). mRNA level of **C** *Igf1r*, **D** *Mapk1* and **E** *Pik3ca* (qPCR, *n* = 4–8). **F**–**I** Densitometric analysis of changes of IGF-1R pathway in response to STZ. **F** Combined analysis of IGF-1R and downstream mediators. Relative to GAPDH reference protein. The protein level of **G** IGF-1R (rel. to GAPDH), **H** pERK1/2 (rel. to total ERK1/2), and **I** p-AKT (rel. to total AKT), (western blot, densitometric analysis of five independent western blot repetitions, *n* = 3–4) upon STZ treatment. **J** IGF-1R expression in different cardiac cell types (CMs, SMCs, ECs; shown with arrows) (IF; ⍺-smooth muscle actin (⍺-SMA)/IGF-1R—green, laminin—purple, Cx43—red). Representative histological sections (×63 magnification, scale bar: 10 µm). Data are presented as mean ± SEM. ^#^*p* < 0.05, ^##^*p* < 0.001, ^###^*p* < 0.0001—two-way ANOVA variation; *p < 0.05, **p < 0.01 ***p < 0.001 by two-way ANOVA with Tukey’s post hoc test; ^$^*p* < 0.05, ^$$^*p* < 0.01 by unpaired two-tailed Student’s *t* test. *Igf1r (IGF-1R)* insulin-like growth factor 1 receptor, *Mapk1 (ERK2)* mitogen-activated protein kinase 1, *Pik3ca* PI3 kinase catalytic subunit alpha, *(p)AKT* (phosphorylated) protein kinase B, *(p)ERK1/2* (phosphorylated) extracellular signal-regulated kinase 1/2

Abundant expression of miR-378a was reported previously both in skeletal muscles and the heart [6, 7]. In the current study, we show a significant upregulation of miR-378a-3p (*p* = 0.0477) and miR-378a-5p (*p* = 0.0032) in diabetic condition 8 weeks upon STZ administration (Fig. 2A, B) with its undetectable expression in miR-378−/− cardiac specimens. We further investigated the regulation of IGF-1R signaling recognized as a multi-level target of miR-378a. In non-diabetic cardiac specimens, *Igf1r* (*t*_13_ = 1.880, *p* = 0.0828)*, Mapk1* (ERK2) (t_13_ = 3.494, *p* = 0.0040)*,* and *Pik3ca* (p110⍺) (*t*_13_ = 3.334, *p* = 0.0054)*,* gained statistical tendency/significance in the absence of miR-378a (Fig. 2C–E). The two-way ANOVA analysis revealed the effect of genotype only in the case of *Pik3ca* (*F*_1,22_ = 8.332, *p* = 0.0086, Fig. 2E).

In the combined analysis of IGF-1R pathway, miR-378a knockout in non-diabetic mice enhanced the protein level of IGF-1R and downstream mediators (phospho-ERK1/2 (p-ERK1/2), p-AKT, ERK1/2, AKT) (*t*_28_ = 2.667, *p* = 0.0126*,* Fig. 2F). An increase in IGF-1R (*t*_4_ = 2.384, *p* = 0.0757*,* Fig. 2G) correlated with the pattern of ERK activation (phosphorylation at Thr202/Tyr204) (*p* > 0.1, Fig. 2H), with opposite direction detected for p-AKT (at Ser473) (*t*_4_ = 4.222, *p* = 0.0135, Fig. 2I) suggesting alternative mechanisms involved.

Despite miR-378a upregulation by STZ (Fig. 2A, B), a tendency to increase IGF-1R signaling was also visible in cardiac STZ-treated miR-378a+/+ specimens (*t*_33_ = 1.720, *p* = 0.0949*,* Fig. 2F). Nonetheless, the level of IGF-1R protein was comparable between control and STZ-treated wild-type mice (Fig. 2G). Interestingly, an increase of IGF-1R signaling observed in non-diabetic miR-378a−/− mice (vs. miR-378a+/+) or upon STZ treatment of miR-378a+/+ mice (vs. miR-378a+/+) was significantly abolished in STZ-treated miR-378a−/− specimens as analyzed by two-way ANOVA (Fig. 2F, G). No such pattern was visible for pERK1/2 (Fig. 2H).

Both, in miR-378a+/+ and miR-378a−/− cardiac tissue sections, IGF-1R expression colocalized with ⍺-smooth muscle actin (⍺-SMA)-positive blood vessels and connexin 43 (Cx43)-positive CMs (Fig. 2J). Considering that miR-378a expression is selective to CMs [7], the presence and action of IGF-1R in other cardiac cell types or the involvement of other rescue mechanisms in the absence of miR-378a should also be considered.

### High glucose increases miR-378a level and promotes pro-hypertrophic IGF-1R pathway in hiPSC-CMs

Due to difficulties with obtaining cardiac muscle material from humans, we harnessed hiPSC-CMs to examine the effect of high glucose (HG) on human CMs. hiPSC-CMs genetically engineered with CRISPR/Cas9 system (hiPSC-CM miR378a+/+ vs. hiPSC-CM miR378a−/−) as reported by us formerly [11], were used to examine the effect of miR378a deprivation.

**Fig. 3:**
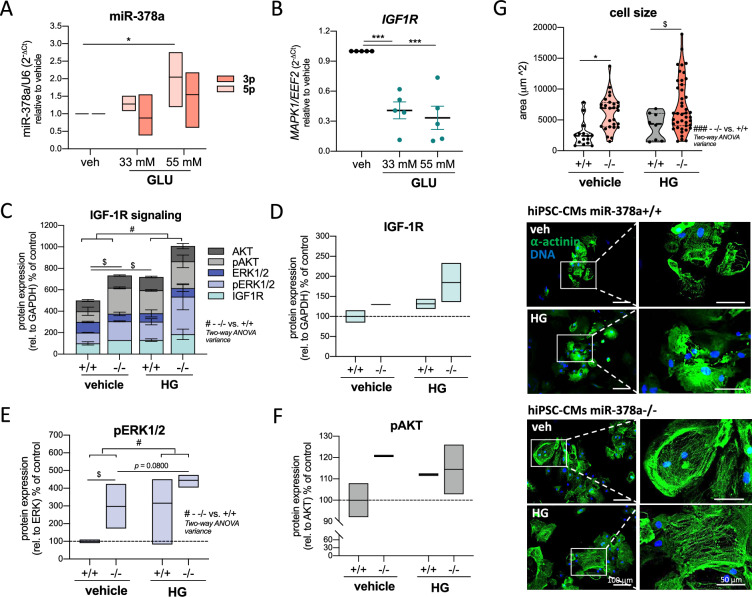
High glucose increases miR-378a expression and stimulates pro-hypertrophic pathways in hiPSC-CMs. miR-378a+/+ hiPSCs and miR-378a−/− hiPSCs were differentiated to CMs and treated with glucose (GLU) for 48 h. Mannitol (55 mM) was used as a vehicle. The expression of **A** miR-378a-3p and -5p (LNA miRCURY qPCR, *n* = 4) and **B** *IGF1R* mRNA level (qPCR, *n* = 5). **C**–**F** Densitometric analysis of changes of IGF-1R pathway in response to glucose. **C** Combined analysis of IGF-1R and downstream mediators. Relative to GAPDH reference protein. **D** Protein level of IGF-1R (rel. to GAPDH), **E** pERK1/2 (rel. to total ERK1/2) and **F** p-AKT (rel. to total AKT), (western blot, densitometric analysis, *n* = 2–4). **G** Quantitative analysis of cell area (*n* = 15/27/8/43) and representative pictures of hypertrophic growth of miR-378a−/− hiPSC-CMs under control conditions and 55 mM glucose (HG) (IF; ⍺-actinin—green), representative images (×200 magnification). Data are presented as mean ± SEM. ^#^*p* < 0.05—two-way ANOVA variation; *p < 0.05, ***p < 0.001 by two-way ANOVA with Tukey’s post hoc test; ^$^*p* < 0.05 by unpaired two-tailed Student’s *t* test. *GLU* glucose, *HG* high glucose, *hiPSC-CMs* human induced pluripotent stem cell-derived cardiomyocytes, *IGF1R (IGF-1R)* insulin-like growth factor 1 receptor, *(p)AKT* (phosphorylated) protein kinase B, *(p)ERK1/2* (phosphorylated) extracellular signal-regulated kinase 1/2

In response to HG (55 mM—fivefold over normal 11 mM culture condition) miR-378a-5p level significantly increased in hiPSC-CMs miR378a+/+ as verified by one-way ANOVA (*F*_2,9_ = 5.473, *p* = 0.0278, Fig. 3A). The same, but less pronounced pattern was detected in case of miR-378a-3p (*F*_2,9_ = 2.146, *p* = 0.1730). Accompanying substantial decline in *IGF1R* mRNA (*F*_2,12_ = 19.31, *p* = 0.0002, Fig. 3B) and downstream *MAPK1* (*F*_2,15_ = 14.65, *p* = 0.0003, Suppl. Figure 1A) was detected. In contrast, at the protein level, in combined analysis of IGF-1R and downstream mediators (p-ERK1/2, p-AKT, ERK1/2, AKT) HG stimulated IGF-1R signaling in control cells (*t*_27_ = 2.636, *p* = 0.0137) and in miR-378a-deficient hiPSC-CMs as analyzed by two-way ANOVA (*F*_1,54_ = 6.056, *p* = 0.0171, Fig. 3C). The same although statistically insignificant pattern was visible for IGF-1R protein (*F*_1,4_ = 2.71, *p* = 0.1748, Fig. 3D). In miR-378a−/− hiPSC-CMs (vs. miR-378a+/+) we detected increased IGF-1R signaling (*t*_30_ = 2.355, *p* = 0.252, Fig. 3C), p-ERK1/2 (*t*_6_ = 3.100, *p* = 0.0211, Fig. 3E) and p-AKT (*t*_2_ = 2.609, *p* = 0.1208, Fig. 3F) proteins. A synergistic increase of p-ERK1/2 was detected in miR-378a-deficient cells upon HG stimulation (*t*_5_ = 2.191, *p* = 0.0800, Fig. 3E) with the same but insignificant pattern seen for combined IGF-1R signaling (*t*_27_ = 1.616, *p* = 0.1176, Fig. 3C).

The activation of the pro-hypertrophic pathway in miR-378a−/− hiPSC-CMs was reflected by the hypertrophic phenotype of those cells. The effect of miR-378a knockout was detected by two-way ANOVA for the genotype × cell area interaction (*F*_1,89_ = 14.85, *p* = 0.0002, Fig. 3G, Suppl. Figure 1D). Specifically, cell area increased in the absence of miR-378a in control conditions (*p* = 0.0132) and under HG stimulation (*t*_49_ = 2.067, *p* = 0.0440) (Fig. 3G) with no apparent glucose effect in any of the genotypes (*p* > 0.1). The cardiac identity was confirmed by the presence of ⍺-actinin in both miR-378a+/+ and miR-378a−/− cells (Fig. 3G).

### STZ stimulates pro-hypertrophic cardiac phenotype in miR-378a-independent manner

**Fig. 4:**
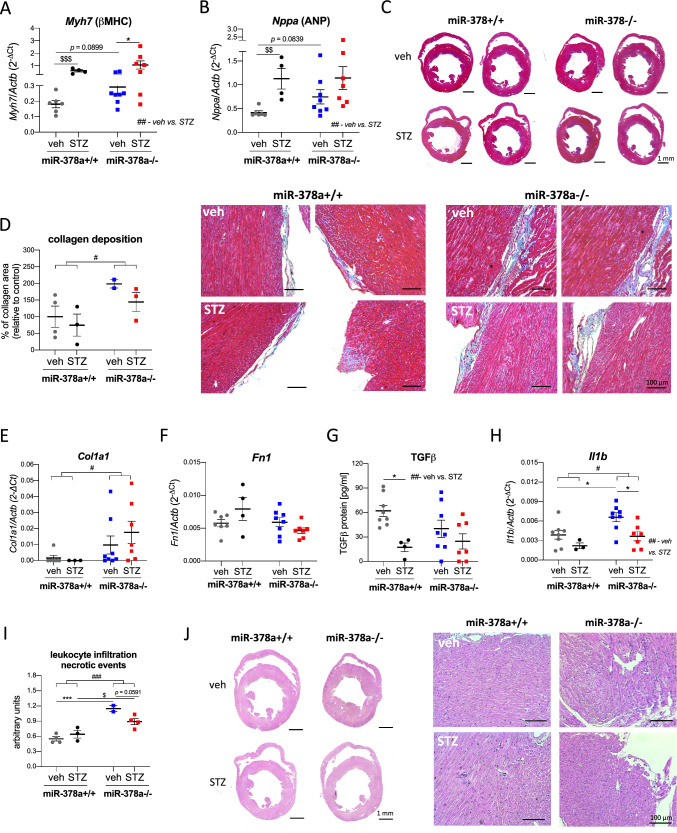
STZ stimulates pro-hypertrophic cardiac phenotype in miR-378a-independent manner. Heart lysates/cardiac histological sections were obtained from either control or STZ-treated miR-378a+/+ and miR-378a−/− mice. Relative expression of **A** myosin heavy chain 7 (*Myh7/*ß-MHC) and **B** natriuretic peptide A (*Nppa/*ANP*)* in the heart lysates, qPCR (*n* = 4–8). **C** Representative cardiac sections and **D** semiquantitative analysis of collagen deposition (Masson’s trichrome staining) (*n* = 2–4). Relative expression of **E** collagen type 1 alpha 1 chain (*Col1a1*) (qPCR), **F** fibronectin 1 (*Fn1*), **G** transforming growth factor-beta (TGF-ß) (ELISA), and **H** interleukin 1 beta (*Il1b*) (qPCR) in cardiac lysates (*n* = 4-8). **I** Semi-quantitative analysis and **J** representative cardiac sections of leukocyte infiltration and necrotic events (H&E staining) (*n* = 2-4). Data are presented as mean ± SEM. ^#^*p* < 0.05, ^##^*p* < 0.001, ^###^*p* < 0.0001—two-way ANOVA variation; *p < 0.05, ***p < 0.001 by two-way ANOVA with Tukey’s post hoc test; ^$^*p* < 0.05, ^$$^*p* < 0.01, ^$$$^*p* < 0.001 by unpaired two-tailed Student’s *t* test.

β-myosin heavy chain (β-MHC), atrial natriuretic peptide (ANP), and brain natriuretic peptide (BNP) are recognized biomarkers of cardiac hypertrophy. A tendency to increase *Myh7* (β-MHC) (*p* = 0.0899, Fig. 4A) and *Nppa* (ANP) (*p* = 0.0839, Fig. 4B) was noticed in the absence of miR-378a in non-diabetic mice. STZ increased *Myh7* (*F*_1,21_ = 9.185, *p* = 0.0064, Fig. 4A) and *Nppa* (*F*_1,21_ = 8.821, *p* = 0.0073, Fig. 4B) in wild type and miR-378a−/− mice, with no difference in *Nppb* (BNP) level (Suppl. Figure 2A). The extent of fibrotic lesions (*F*_1,8_ = 6.737, *p* = 0.0318, Fig. 4C, D) and collagen type I alpha 1 chain (*Col1a1*) expression (*F*_1,20_ = 4.398, *p* = 0.0489, Fig. 4E) were more pronounced in miR-378a−/− hearts (vs. miR-378a+/+), while not affected by diabetic conditions. No significant changes in fibronectin 1 (*Fn1*) mRNA were detected between tested groups (Fig. 4F).

Transforming growth factor-β (TGF-β) is a critical regulator of scar formation and tissue repair promoting not only myofibroblast phenotype but also cardiomyocyte apoptosis and hypertrophy [19]. We detected a decrease of TGF-β in STZ-induced diabetic hearts (*F*_1,23_ = 10.30, *p* = 0.0039), particularly in miR-378a+/+ mice (*p* = 0.0232) (Fig. 4G). Such data point to TGF-β-independent pro-fibrotic effects of miR-378a deficiency in this experimental setting.

**Fig. 5:**
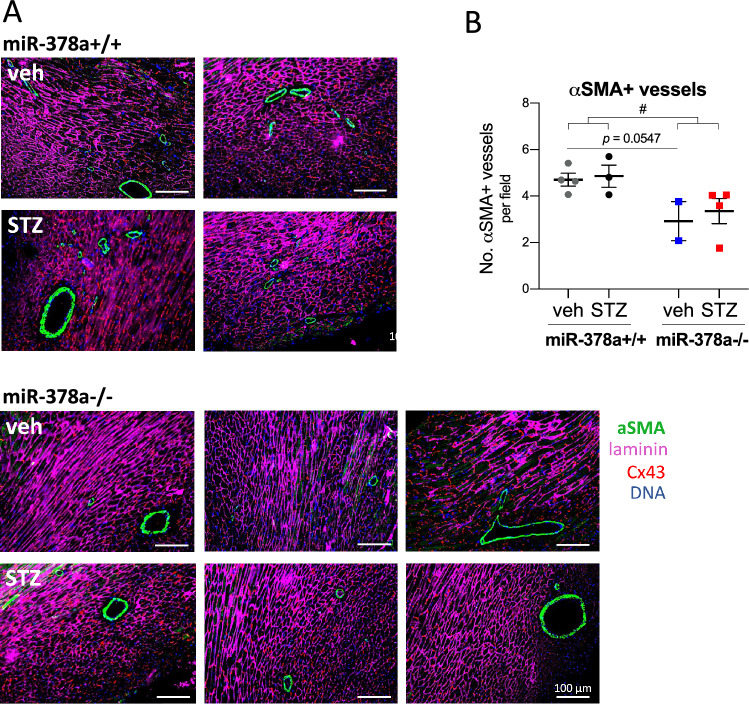
MiR-378a deficiency decreases the number of ⍺-SMA-positive blood vessels independently of STZ. **A** Representative cardiac sections and **B** quantitative analysis of ⍺-SMA-positive vessels (IF; ⍺-SMA—green, laminin—purple, Cx43—red) (*n* = 2–4). Data are presented as mean ± SEM. ^#^*p* < 0.05—two-way ANOVA variation. *⍺-SMA* ⍺-smooth muscle actin, *Cx43* connexin 43

An upregulation of pro-inflammatory interleukin 1 beta (*Il1b*) was detected in the absence of miR-378a (*F*_1,22_ = 7.261, *p* = 0.0132), with a significant increase in non-diabetic mice (*p* = 0.0313) reversed by STZ (*p* = 0.0235) (Fig. 4H). Analysis of tissue morphology revealed augmented inflammatory infiltration and necrosis in miR-378a−/− hearts (*F*_1,9_ = 44.63, *p* < 0.0001, Fig. 4I, J), with a significant increase in both vehicle - (*p* = 0.0007) and STZ-treated specimens (*p* = 0.0498). No significant differences were observed in *Tnfa* and* Il6* (Suppl. Figure 2B, C) as well as serum LDH or CK activity (Suppl. Figure 2D, E). In addition, miR-378a deficiency decreased the number of ⍺-SMA-positive blood vessels as shown by a two-way ANOVA for the genotype x No. blood vessels interaction (*F*_1,9_ = 10.10, *p* = 0.0112) with no significant impact of diabetic component (*F*_1,9_ = 0.3229, *p* = 0.5838) (Fig. 5A, B). No genotype-dependent differences were seen in VEGF and FGF2 expression in cardiac lysates (Suppl. Figure 3).

### miR-378a does not affect STZ-induced changes in cardiac function parameters

**Fig. 6:**
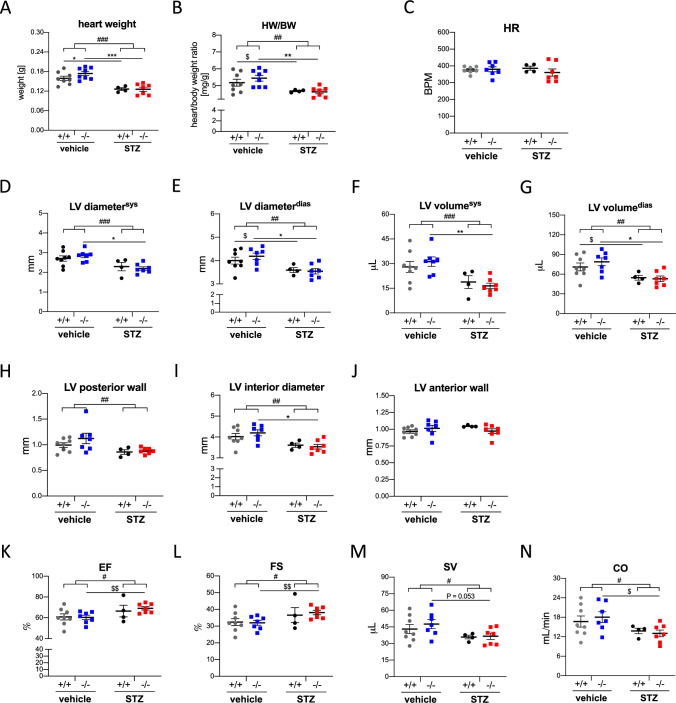
miR-378a does not affect STZ-induced changes in cardiac parameters. **A** Heart weight and **B** ratio of heart weight to body weight (HW/BW) 8 weeks upon STZ treatment. **C** Heart rate (HR). Left ventricular (LV) **D**, **E** diameter and **F**, **G** volume at systole (sys) and diastole (dias), **H** LV posterior wall, **I** LV interior diameter, **J** LV anterior wall, **K** LV ejection fraction (EF), **L** LV fractional shortening (FS), **M** stroke volume (SV), and **N** cardiac output (CO). In vivo high-resolution ultrasound imaging (*n* = 4–8). Data are presented as mean ± SEM. ^#^*p* < 0.05, ^##^*p* < 0.001, ^###^*p* < 0.0001—two-way ANOVA variation; *p < 0.05, **p < 0.01, ***p < 0.001 by two-way ANOVA with Tukey’s post hoc test; ^$^*p* < 0.05, ^$$^*p* < 0.01 by unpaired two-tailed Student’s *t* test

Cardiac fibrotic changes present in miR-378a−/− mice suggest increased cardiac stiffness that could lead to cardiac diastolic dysfunction. Nonetheless, genotype-dependent histological differences were not reflected by in vivo high-resolution ultrasound imaging. The heart weight (*F*_1,23_ = 35.77, *p* < 0.0001, Fig. 6A) and the ratio of heart weight versus body weight (HW/BW) (*F*_1,23_ = 13.26, *p* = 0.0014, Fig. 6B) diminished both in miR-378a+/+ and in miR-378a−/− mice upon STZ treatment. No changes in heart rate were detected between tested groups (Fig. 6C). Decreased LV systolic (*F*_1,22_ = 16.10, *p* = 0.0006, Fig. 6D) and diastolic diameter (*F*_1,22_ = 13.40, *p* = 0.0014, Fig. 6E) as well as LV systolic (*F*_1,22_ = 15.04, *p* = 0.0008, Fig. 6F) and diastolic volume (*F*_1,22_ = 13.43, *p* = 0.0014, Fig. 6G) were noticed in STZ-induced diabetic hearts of both genotypes. In line with that, we detected a drop in LV posterior wall (LVPW) thickness (*F*_1,22_ = 7.980, *p* = 0.0099, Fig. 6H) and interior diameter (LVID) (*F*_1,22_ = 13.74, *p* = 0.0012, Fig. 6I) upon STZ treatment with no changes in LV anterior wall (LVAW, Fig. 6J). Furthermore, although STZ, independently of genotype, increased LV ejection fraction (EF) (*F*_1,22_ = 6.002, *p* = 0.0227, Fig. 6K) and fractional shortening (FS) (*F*_1,22_ = 5.491, *p* = 0.0286, Fig. 6L) as revealed by two-way ANOVA, it decreased overall heart function as shown by a decrease in stroke volume (SV) (*F*_1,22_ = 5.352, *p* = 0.0304, Fig. 6M) and cardiac output (CO) (*F*_1,22_ = 6.263, *p* = 0.0203, Fig. 6N). Thus, neither STZ-driven miR-378a upregulation in wild-type mice nor miR-378a deficiency changed a drop in overall diabetic heart function.

## Discussion

Here, we report the upregulation of miR-378a in cardiac hyperglycemic milieu and HG-treated human hiPSC-CMs. Although we underlined the involvement of miR-378a in maintaining basal cardiac structural integrity and vascularization, we questioned the importance of endogenous miR-378a in the development of DCM. Results point to miR-378a-independent hyperglycemia-driven cardiac hypertrophy and associated dysfunction.

STZ administration is well described, reproducible model of type 1 diabetes, manifested by insulitis, reduction in insulin secretion capacity, and hyperglycemia, although due to its development in the absence of T- and B cell-contribution not perfectly resembles autoimmune human condition [18]. Nonetheless, the immunomodifying effects of STZ were described [20] and confirmed in our study by blood lymphopenia and monocytopenia. Acute hyperglycemia itself seems to be a direct cause of immunosuppression growing in any diabetic environment [20]. It was previously reported that STZ-elicited diabetes in rodents is related to progressive weight loss [14, 21, 22] and decreased heart weight with an unchanged HW/BW ratio between non-diabetic and STZ-treated groups [14, 22]. In line with that in the current study, we showed a genotype-independent drop in body and heart weight, but also in HW/BW ratio.

CM hypertrophy is initiated in response to increased cardiac workload (exercise) or injury acting as a compensatory response to maintain cardiac output [3]. Pathological hypertrophy is associated with fibrosis and myofiber disarray [23] eventually increasing cardiac stiffness and compromising heart function [19]. IGF-1 as a critical mediator of physiological hypertrophy acts against cardiac dysfunction [24, 25]. In a diabetic environment, IGF-1/IGF-1R axis was shown to ameliorate the pathophysiological progress of DCM through antioxidative and anti-inflammatory processes [12–14]. However, any link to miR-378a has not been reported so far.

In the current study, STZ-induced diabetes did not change significantly cardiac IGF-1R expression. It is in agreement with previous studies that also showed comparable [14, 26] or even decreased [27] IGF-1R protein levels in STZ-treated rodents. It could be explained by the upregulation of miR-378a observed by us in murine diabetic hearts, and in human model, HG-treated hiPSC-CMs. Our data expand and verify previous studies that showed an insignificant increase of miR-378a in diabetic whole heart tissue upon multiple low-dose STZ injections [28] and are in line with reported upregulation of miR-378a in LV specimens of mice given a single high-dose injection of STZ [5].

We previously reported that miR-378a-deficient hiPSC-CMs exhibit hypertrophic growth with the involvement of NFATc3 (nuclear factor of activated T cells c3) and the activation of ERK and AKT kinases [11]. In the current study, we verified the hypertrophic phenotype of miR-378a−/− hiPSC-CMs both in control conditions and under HG stimulation with associated increase of IGF-1R signaling, and p-ERK1/2 and p-AKT proteins. The level of biomarkers of cardiac hypertrophy, β-MHC and ANP, increased in miR-378a deficiency (in non-diabetic mice) and STZ-treated miR-378a+/+ mice. This correlated with the pattern of pro-hypertrophic IGF-1R pathway activation in respective murine cardiac specimens as well as miR-378−/− hiPSC-CMs and HG-cultured miR-378a+/+ hiPSC-CMs that also showed increased size, and suggests miR-378a independent hyperglycemia-promoted hypertrophy. Interestingly, STZ-dependent increase in hypertrophic markers was not reflected by cardiac weight. It could be explained by the early stage of cardiac hypertrophy detected by markers expression in cardiac lysates but not yet reflected by enhanced heart weight.

Moreover, we detected a synergistic upregulation of the IGF-1R pathway and cell growth (although not significant) in miR-378a-deficient HG-treated hiPSC-CMs. Thus, miR-378a upregulation in HG conditions could be considered as a compensatory mechanism to decrease pro-hypertrophic pathways. Nonetheless, at the tissue level, mir-378a does not appear to be a key regulator of the IGF-1R pathway in the diabetic heart, as even in its absence this pathway is blocked. The involvement of other rescue mechanisms in the absence of miR-378a in the hyperglycemic milieu needs to be further investigated. The presence and action of IGF-1R in other cardiac cell types should also be considered.

Myocardial fibrosis associated with the accumulation of extracellular matrix proteins constitutes a critical component of DCM pathology [2] and was reported in STZ-diabetic animal models [12, 14, 22]. However, in our experimental setting STZ-driven hyperglycemia although promoted myocardial hypertrophy, did not affect the extent of fibrotic lesions and collagen expression which requires further analysis and constitutes the limitation of the current study.

The impact of miR-378a on fibrotic structural changes seems to be tissue-dependent and affected by disease states [17, 29, 30]. Previous data point to anti-fibrotic effects of miR-378a in the murine model of pressure overload [8, 10]. Compensation for miR-378 downregulation by AAV9-miR378 administration diminished cardiac fibrosis and increased cardiac contractility [10]. In the current work we showed that collagen deposition increased in miR-378a−/− hearts of both non-diabetic controls and upon STZ treatment. Pro-fibrotic effects of miR-378a deficiency were not mediated by TGF-β, but an STZ-dependent drop of TGF-β was detected in wild-type animals where miR-378a was increased. Nonetheless, bioinformatic screening did not find *Tgfb* as a direct target of miR-378a-3p or miR-378a-5p, hence the effect requires further mechanistical investigation.

The 8-week study period upon STZ treatment was shown to be sufficient to induce cardiac dysfunction in C57BL6J mice [14, 22]. The pattern of diabetes-related decrease of end-diastolic dimension and end-systolic dimension with increased LVPW thickness was demonstrated previously by Huynh et al. [14] although statistically insignificant. In the current study, we confirmed genotype-independent STZ-driven decrease of end-systolic and end-diastolic LV diameter and LV volume while showing a drop in stroke volume (SV) and cardiac output (CO) in diabetic conditions. It is in agreement with diastolic dysfunction shown by Huynh et al. using Doppler echocardiography and LV catheterization that was consistent with increased fibrosis [14]. Despite that, in our model in both miR-378a+/+ and miR-378a−/− mice, we detected increased contractility assessed by LV EF and FS upon STZ treatment. Although diabetes-related increase of FS was also detected by others [14] it requires further investigation.

Here, we examined for the first time the involvement of miR-378a in diabetic heart function. Neither STZ-driven miR-378a upregulation in wild-type mice nor miR-378a deficiency changed the pattern of decreased SV and CO observed in vivo by high-resolution ultrasound imaging. MiR-378a-independent compromised diabetic heart function may be related to ERK signaling and enhanced hypertrophic markers in STZ-treated animals. On the other hand decreased AKT activity in cardiac tissue shown in diabetic hearts of mice that were given a single injection of STZ [31] and also detected in the current study, could be involved. Despite the involvement of AKT in cardiac hypertrophy, the correlation of improvement of cardiac function and structure with elevated AKT activity was reported in diabetic IGF-1R transgenic mice as compared to nontransgenic diabetic mice, suggesting the protective effects of IGF-1R signaling in diabetes [14]. In parallel, in the same study elevated ventricular AKT phosphorylation was also detected in nontransgenic diabetic animals compared to non-diabetic controls [14]. Thus, one may consider disturbed balance between physiological and pathological cardiac hypertrophy and underlying mechanisms that could determine the diabetic phenotype.

Having in mind a progressive increase of cardiac miR-378a expression from birth to aged mice [7] the effect of miR-378a deficiency might not be severe enough to affect overall cardiac function in used in our study of 10–13-week-old mice. In line with this explanation, in 25-week-old *Db/Db* mice, a model of type 2 diabetes, miR-378a loss was shown to improve cardiac function [32] which further enhances the hypothesis of the involvement of other rescue mechanisms in the absence of miR-378a in hyperglycemic milieu still to be investigated.

In summary, this work proves hyperglycemia/HG-driven upregulation of miR-378a expression in the murine heart and novel human CM model. MiR-378a deficiency impaired basal cardiac structural integrity, but changes in mir-378a levels seem to be compensated in the diabetic milieu by other mechanisms regulating the IGF-1R pathway, which questions the importance of endogenous miR-378a in this disease model.

### Supplementary Information

Below is the link to the electronic supplementary material.

Suppl. Fig. 1. The regulation of the IGF-1R pathway by high glucose in cardiac lysates of miR-378a+/+ and miR-378a-/- mice and hiPSC-CMs. (A) Heart lysates were obtained from either control or STZ-treated miR-378a+/+ and miR-378a-/- mice. Exemplary western blots of five independent repetitions. (B–C) miR-378a+/+ hiPSCs and miR-378a-/- hiPSCs were differentiated into cardiomyocytes and treated with high glucose (HG) for 48 h. Mannitol (55 mM) was used as a vehicle. (B) The MAPK1 mRNA level (qPCR, n=6) and (C) protein level of IGF-1R and downstream mediators (western blots) after 48-hour high glucose (HG) treatment. Data are presented as mean ± SEM. ** p < 0.01, *** p < 0.001 by one-way ANOVA with Tukey’s post hoc test. *hiPSC-CMs* human induced pluripotent stem cells-derived cardiomyocytes, *IGF1R (IGF-1R)* insulin-like growth factor 1 receptor, *MAPK1* mitogen-activated-protein kinase 1, *(p)AKT* (phosphorylated) protein kinase B, *(p)ERK1/2 * (phosphorylated) extracellular signal-regulated kinase 1/2 (PDF 3370 KB)

Suppl. Fig. 2. The effect of miR-378a deficiency and STZ treatment on cytokine release in the heart and serum muscle damage markers. (A–C) Heart lysates were obtained from either control or STZ-treated miR-378a+/+ and miR-378a-/- mice. The expression of (A) natriuretic peptide A (Nppa/ANP), (B) tumor necrosis factor alpha (Tnfa), and (C) interleukin 6 (Il6) in heart lysates of miR-378a+/+ and miR-378a-/- mice 8 weeks upon STZ treatment, qPCR (n=3-8). (D) Lactate dehydrogenase (LDH) and (E) creatine kinase (CK) activity in serum. Activity assays. Data are presented as mean ± SEM. $—p < 0.05 by Student’s *t* test. (PDF 185 KB)

Suppl. Fig.3. The effect of miR-378a deficiency and STZ treatment on VEGF and FGF2 expression in the heart. Heart lysates were obtained from either control or STZ-treated miR-378a+/+ and miR-378a-/- mice. (A) The mRNA level of vascular endothelial growth factor (Vegf) in heart lysates upon 8-week STZ treatment, qPCR (n=4-8). The protein level of (B) VEGF and (C) fibroblasts growth factor 2 (FGF2) in heart lysates upon STZ treatment, ELISA (n=4-8). Data are presented as mean ± SEM. #—p < 0.05—two-way ANOVA variation. (PDF 105 KB)

## Data Availability

The datasets generated during and/or analyzed during the current study are available from the corresponding author upon reasonable request.

## Abbreviations

⍺-SMA: ⍺-Smooth muscle actin
AKT: Protein kinase B
ANP (*Nppa*): Natriuretic peptide A
β-MHC (*Myh7)*: β-Myosin heavy chain
BNP (*Nppb)*: Natriuretic peptide B
CMs: Cardiomyocytes
*Col1a1*: Collagen type 1 alpha 1 chain
Cx43: Connexin 43
DCM: Diabetic cardiomyopathy
ERK: Extracellular signal-regulated kinase
FGF2: Fibroblast growth factor 2
*Fn1*: Fibronectin 1
GSK-3β: Glycogen synthase kinase-3 beta
HG: High glucose
hiPSCs: Human induced pluripotent stem cells
IGF-1: Insulin-like growth factor 1
IGF-1R: IGF-1 receptor
*Il1b*: Interleukin 1 beta
MAPK1 (ERK2): Mitogen-activated protein kinase 1
NFATc3: Nuclear factor of activated T cells c3
PGC-1β: Peroxisome proliferator-activated receptor gamma coactivator-1 beta
STZ: Streptozotocin
TGF-ß: Transforming growth factor-beta
*Tnfa*: Tumor necrosis factor alpha
VEGF: Vascular endothelial growth factor
