# Supplementary figures and images for: Compromised diabetic heart function is not affected by miR-378a upregulation upon hyperglycemia
Source: Pharmacol Rep. 2023 Oct 18;75(6):1556–70. doi: 10.1007/s43440-023-00535-8 (PMC10661816; doi:10.1007/s43440-023-00535-8)

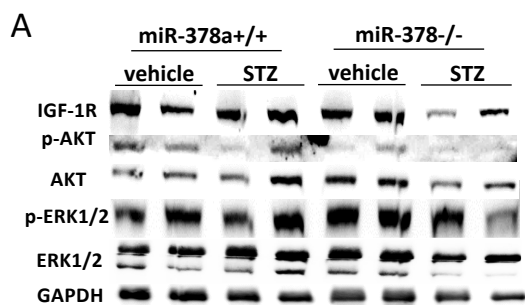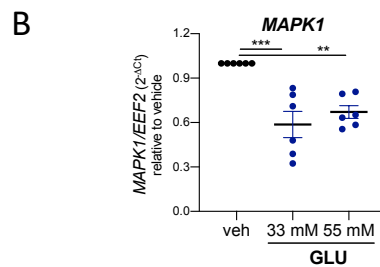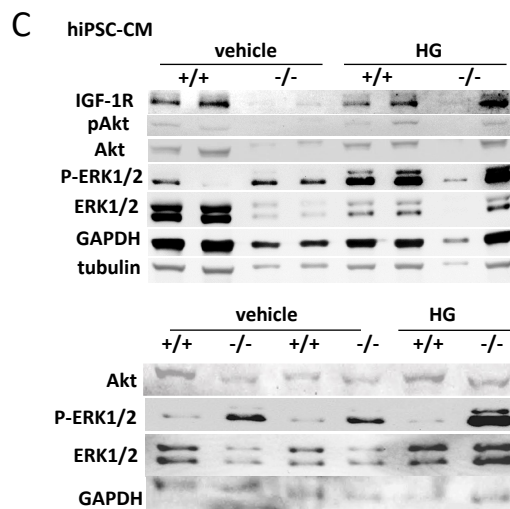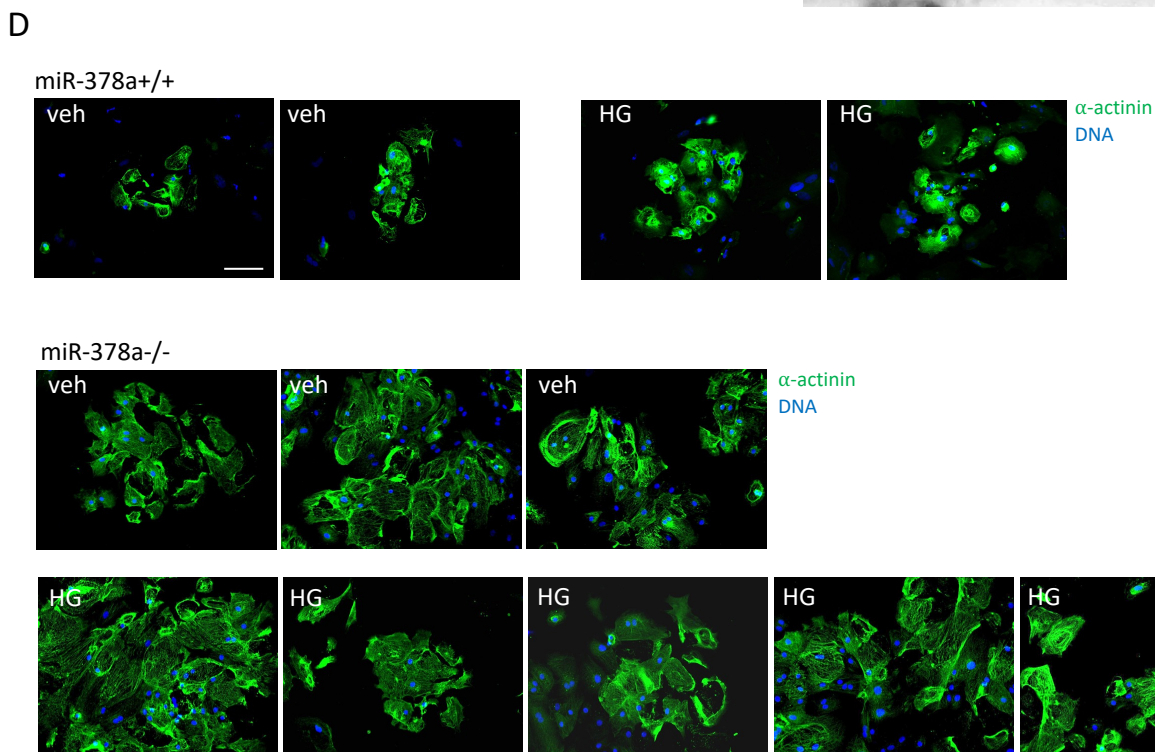

Supplement: Supplementary file 1 — Suppl. Fig. 1. The regulation of the IGF-1R pathway by high glucose in cardiac lysates of miR-378a+/+ and miR-378a-/- mice and hiPSC-CMs. (A) Heart lysates were obtained from either control or STZ-treated miR-378a+/+ and miR-378a-/- mice. Exemplary western blots of five independent repetitions. (B–C) miR-378a+/+ hiPSCs and miR-378a-/- hiPSCs were differentiated into cardiomyocytes and treated with high glucose (HG) for 48 h. Mannitol (55 mM) was used as a vehicle. (B) The MAPK1 mRNA level (qPCR, n=6) and (C) protein level of IGF-1R and downstream mediators (western blots) after 48-hour high glucose (HG) treatment. Data are presented as mean ± SEM. ** p < 0.01, *** p < 0.001 by one-way ANOVA with Tukey’s post hoc test. hiPSC-CMs human induced pluripotent stem cells-derived cardiomyocytes, IGF1R (IGF-1R) insulin-like growth factor 1 receptor, MAPK1 mitogen-activated-protein kinase 1, (p)AKT (phosphorylated) protein kinase B, (p)ERK1/2 (phosphorylated) extracellular signal-regulated kinase 1/2 (PDF 3370 KB) [file 43440_2023_535_MOESM1_ESM.pdf]

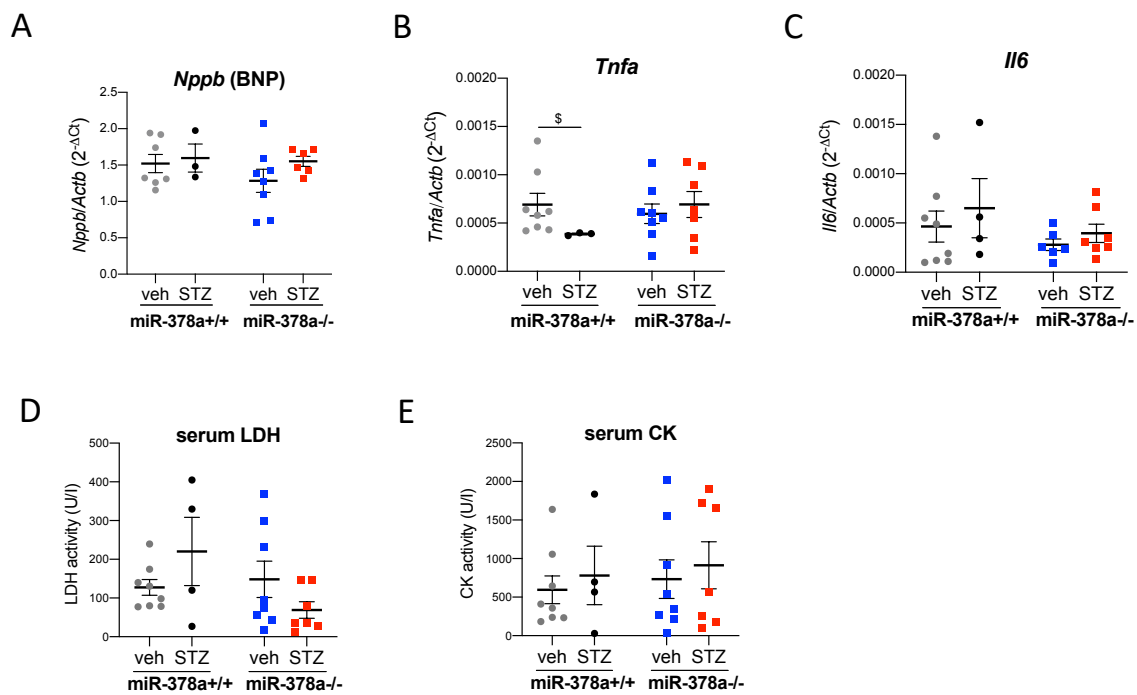

Supplement: Supplementary file 2 — Suppl. Fig. 2. The effect of miR-378a deficiency and STZ treatment on cytokine release in the heart and serum muscle damage markers. (A–C) Heart lysates were obtained from either control or STZ-treated miR-378a+/+ and miR-378a-/- mice. The expression of (A) natriuretic peptide A (Nppa/ANP), (B) tumor necrosis factor alpha (Tnfa), and (C) interleukin 6 (Il6) in heart lysates of miR-378a+/+ and miR-378a-/- mice 8 weeks upon STZ treatment, qPCR (n=3-8). (D) Lactate dehydrogenase (LDH) and (E) creatine kinase (CK) activity in serum. Activity assays. Data are presented as mean ± SEM. $—p < 0.05 by Student’s t test. (PDF 185 KB) [file 43440_2023_535_MOESM2_ESM.pdf]

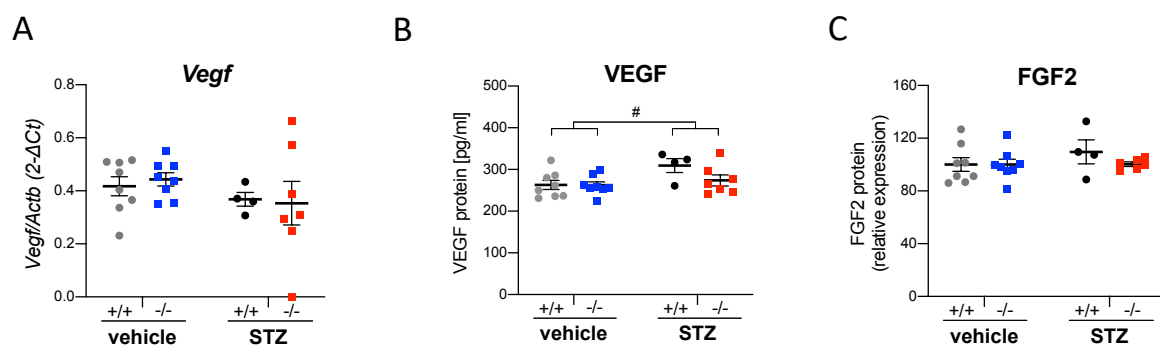

Supplement: Supplementary file 3 — Suppl. Fig.3. The effect of miR-378a deficiency and STZ treatment on VEGF and FGF2 expression in the heart. Heart lysates were obtained from either control or STZ-treated miR-378a+/+ and miR-378a-/- mice. (A) The mRNA level of vascular endothelial growth factor (Vegf) in heart lysates upon 8-week STZ treatment, qPCR (n=4-8). The protein level of (B) VEGF and (C) fibroblasts growth factor 2 (FGF2) in heart lysates upon STZ treatment, ELISA (n=4-8). Data are presented as mean ± SEM. #—p < 0.05—two-way ANOVA variation. (PDF 105 KB) [file 43440_2023_535_MOESM3_ESM.pdf]
